# The association of prenatal amniotic sex hormones and digit ratio (2D:4D) in children aged 5 to 70 months: A longitudinal study

**DOI:** 10.1371/journal.pone.0282253

**Published:** 2023-03-23

**Authors:** Luisa Ernsten, Lisa M. Körner, Marie Luisa Schaper, Judith Lawrenz, Gareth Richards, Martin Heil, Nora K. Schaal

**Affiliations:** 1 Department of Experimental Psychology, Heinrich-Heine-University, Düsseldorf, Germany; 2 Department of General Pediatrics, Neonatology and Pediatric Cardiology, University Children’s Hospital, Medical Faculty, Heinrich-Heine-University, Düsseldorf, Germany; 3 School of Psychology, Faculty of Medical Sciences, Newcastle University, Newcastle upon Tyne, United Kingdom; Northumbria University, UNITED KINGDOM

## Abstract

The sex difference of the 2D:4D digit ratio (female > male)–a proposed marker for prenatal testosterone exposure—is well established. Studies suggest it already exists in utero and is of moderate effect size in adulthood. However, evidence for the claim that 2D:4D reflects prenatal androgen action is limited, and the sex difference may exhibit lability during childhood. In the present study, 244 mothers were recruited in the course of an amniocentesis examination (performed between gestational weeks 14 and 18). Prenatal testosterone (T) and estradiol (E) levels were determined from amniotic fluid for boys and girls. The majority (97.4%, *n* = 114) of available female T levels (*n* = 117) were found below the level of quantification. Therefore, only male amniotic fluid data (*n* = 117) could be included for the analysis of associations between amniotic sex hormones (T levels and T to E ratio (T/E)) and 2D:4D. The families were then invited to each of the five consecutive follow-ups (ages: 5, 9, 20, 40, and 70 months) where children’s 2D:4D was measured for both hands. The alternative marker D_[r-l]_ reflects the directional asymmetry of 2D:4D (right subtracted by left 2D:4D) and was subsequently calculated as an additional measure for prenatal T exposure. No significant correlations between amniotic T or the T/E ratio (measured between week 14 and 18 of gestation) with 2D:4D respectively D_[r-l]_ were observed for any time point. There was a significant sex difference (females > males) and a significant age effect with moderate correlations of 2D:4D between time points. 2D:4D increased between 20 and 40 months and between 40 and 70 months of age. The findings raise questions regarding the applicability of 2D:4D as a marker for prenatal androgen action and are discussed in terms of the reliability of obtained digit ratio data as well as in terms of the developmental timing of amniocentesis.

## Introduction

Since Manning, Scutt, Wilson, and Lewis-Jones [1] suggested the sexually differentiated ratio between the second and fourth digit length (2D:4D or digit ratio) as a marker for prenatal testosterone (T) exposure, a vast number of studies have investigated relationships between 2D:4D and different human behaviors and traits (for reviews see [2–6]). The fascination stems from the assumption that prenatal T has organizing effects on the brain and in turn affects sex-specific behavior later in life [7]. Considering the difficulties inherent with measuring prenatal sex hormones, a convenient and easily obtainable proxy such as 2D:4D is particularly appealing. However, questions remain regarding its validity.

The sex difference in 2D:4D (female > male) is well established [8] and studies of aborted fetuses suggest that it already exists in utero [9, 10]. People characterized by atypical prenatal hormonal environments have also been reported to exhibit differences in 2D:4D. Notably, individuals with complete androgen insensitivity syndrome (CAIS; 46,XY karyotype, but female phenotype) exhibit feminized 2D:4D [11, 12]. However, the effect sizes reported for the difference between healthy controls and females with CAIS were small. Further, the variance in 2D:4D for these samples does not appear to differ from that of controls, which questions the premise that it is prenatal testosterone acting on the developing tissues that explains the effect [13]. Individuals affected by congenital adrenal hyperplasia (CAH), who are exposed to high levels of androgens during the prenatal period, show masculinized 2D:4D compared to females without CAH [14–16]. However, evidence is inconsistent and the effects sizes are relatively small [14–17]. A recent meta-analysis [16] showed only small to medium sized differences in 2D:4D between CAH patients and healthy controls. Additionally, it was noted that individual studies have typically utilized small sample sizes, and that there has been relatively little research published in this area during the past decade. Other studies have reported that men with Klinefelter syndrome (47,XXY karyotype), i.e. phenotypical males with an additional X chromosome, have a more feminized 2D:4D than unaffected controls [18, 19].

2D:4D has repeatedly been shown to correlate with various behavioral measures [20], developmental conditions [21, 22], and even various types of cancer [23, 24]. However, the validity of 2D:4D as a marker for prenatal testosterone exposure has been questioned [20, 25, 26]. Nonetheless, according to a PubMed search using the keywords “2D:4D” and “digit ratio” there were more than 100 newly published studies from 2020 till today that have investigated associations between 2D:4D and various behaviors, traits, disorders, or diseases, like muscular strength and fitness [5, 27], parental income [28], sexual preferences [29], concentration of steroids like cortisol and vitamin D [30], thyroid disease [31], and migraine in adults [32].

In order to investigate whether 2D:4D is a valid proxy for prenatal androgen levels, studies using a direct measurement of hormonal exposure are required. However, it is challenging to obtain such data in human studies due to obvious ethical considerations. Some researchers have measured prenatal sex hormone exposure from amniotic fluid obtained during the course of medically necessary amniocenteses [7, 33, 34]. The advantage of this is the timing of amniocentesis, which is normally conducted between gestational weeks 14 and 20, a timeframe in which sex differences in amniotic T and fetal serum T are highest [35–38]. Amniocentesis examinations are conducted in cases of suspected chromosomal anomalies or due to other risk factors (e.g., higher maternal age) during the second trimester of pregnancy. As this procedure is associated with a small risk of miscarriage as a consequence of invasive sampling [39, 40], non-invasive first trimester screening techniques have recently replaced many amniocentesis examinations [41]. It has therefore become less feasible to collect amniotic fluid samples, with only a few studies relating such data to subsequent phenotypic outcomes [42].

To date there are only three studies investigating associations between amniotic T and 2D:4D. Lutchmaya, Baron-Cohen, Raggatt, Knickmeyer, and Manning [43] published a prominent study showing that 2D:4D of the right hand of 2-year-old children was negatively correlated with the ratio of amniotic T to amniotic estradiol (E), referred to as the T/E ratio, measured during the second trimester of pregnancy. The sample consisted of 29 mother-infant dyads (18 male infants). Male infants exhibited descriptively lower 2D:4D than female infants, but this sex difference was not statistically significant. No significant association emerged between 2D:4D and T or E. The authors suggested the T/E ratio has an influence on fetal growth, developmental conditions like autism, protection against early onset of breast cancer in women or myocardial infarcts in men, as well as athletic abilities in men. This study is frequently cited as evidence for 2D:4D being a valid marker for prenatal sex hormone exposure, despite its methodological limitations. Notably, the sample size was quite small and correlational analyses were not conducted separately for girls and boys, although sex was controlled for as a covariate in multiple regression analysis [43]. Furthermore, and critically, a replication of the study did not find the proposed negative correlation between prenatal T and E measured in amniotic fluid as well as the T/E ratio between weeks 15 and 20 of gestation and 2D:4D measured after 4.5 years in a sample of 66 children and their mothers [44]. Richards Browne, and Constantinescu [44] found, in contrast to the assumed correlations based on Lutchmaya et al. [43], no associations between T as well as the T/E ratio and 2D:4D for either girls or boys. The authors also examined the difference between right and left 2D:4D (D_[r-l]_). This variable is discussed as an additional measure, with lower values thought to indicate a higher intrauterine androgen exposure [45–47]. However, this variable also showed no association with prenatal androgens [44]. The third amniocentesis study from Ventura, Gomes, Pita, Neto, and Taylor found the expected negative relationship between amniotic T and 2D:4D measured a few days after birth [48]. However, this was present only for the left hand and only in newborn girls (significant correlation for left 2D:4D, statistical trend for right 2D:4D [48]).

Another issue in 2D:4D research is limited evidence of temporal stability, especially in young cohorts. Based on the assumption that 2D:4D is (at least partially) determined by prenatal androgens one can assume that the temporal stability should be high, especially before the onset of puberty. However, a review of relevant studies revealed limited evidence for the temporal stability of 2D:4D, and, moreover, the sex difference in prepubertal cohorts [49]. Longitudinal studies have shown an increase of 2D:4D with age, although with moderate to high correlations between the time points [20, 50–52]. Across these studies, the sex difference in 2D:4D was in general small and did not always reach statistical significance, although the direction (female > male) was always the same [50, 51]. A longitudinal study of 0-2-year-olds (age: 2 weeks, 12 months, 24 months) reported a significant sex effect for 2D:4D measured two weeks after birth as well as a decrease in 2D:4D in the first year and an increase in the second year of life [53]. Interestingly, at 12 and 24 months there were no significant sex differences in 2D:4D. However, cross-sectional studies revealed a sex difference as well as an increase in 2D:4D for children between the ages of 2 and 5 years [54] and in a cohort aged between 5 and 17 years [55].

Manning and Fink [56] have promoted the assumption that 2D:4D remains independent of skeletal growth, with an earlier cross-sectional study [1] reporting a stable sex difference and no significant age effects between cohorts of 2-25-year-old subjects. However, the majority of longitudinal as well as cross-sectional studies do not indicate the temporal stability of 2D:4D in younger cohorts (especially under the age of two), which may be affected by skeletal and overall growth. Studies with older cohorts reveal greater and more stable sex differences (specifically adult and adolescent samples [8]), in a timeframe where the finger lengths remain more or less the same [57].

It becomes apparent that the available literature does not allow precise conclusions on the hypothesized validity and reliability of 2D:4D as a marker of prenatal androgen action. Nonetheless, 2D:4D remains a popular tool used by researchers attempting to investigate the effects of prenatal androgen action. For these reasons, the current study examines amniocentesis data of pregnant women and their offspring’s 2D:4D in a longitudinal design. This approach provides an opportunity to test whether 2D:4D is a valid proxy for prenatal T levels, and further to test its temporal stability within a young cohort. Amniotic fluid was obtained between gestational weeks 14 and 18 in a sample of 244 pregnant women in Germany. The overall levels of amniotic T and E were determined, and the T/E ratio was calculated for further analysis. Unfortunately, data were only available for the male subsample as female T levels were under the limit of detection or quantification. Children’s 2D:4D on both hands was measured at 5, 9, 20, 40, and 70 months of age. With respect to the hypothesis that 2D:4D acts as a marker for prenatal androgen exposure, negative correlations between amniotic T as well as the T/E ratio and 2D:4D were predicted for the male subsample at each time point. As other studies also analyzed a possible association between amniotic T and T/E ratio with D_[r-l]_, this additional variable was included. We also predicted that 2D:4D would show moderate to high correlations between the measurement time points, as well as a stable sex difference (females > males for all time points). Further, based on the available literature, an increase of 2D:4D with age was expected.

## Methods

### Participants

Two-hundred forty-four mothers who underwent amniocentesis at a practice of gynecologists and human genetics (*Praenatal*.*de*) in Düsseldorf, Germany, were recruited between 2010 and 2012. The children were born between January 2011 and February 2013 and were invited to the Department of Experimental Psychology at the University of Düsseldorf, Germany, on five occasions. More specifically, participants took part in follow-up research at the ages of 5 months (T1), 9 months (T2), 20 months (T3), 40 months (T4), and 70 months (T5) as part of another study that also obtained behavioral data published elsewhere [58, 59]. Although every initially recruited family was invited to participate, not every family took part in all consecutive measurement time points resulting in different sample sizes (for mean ages, sample sizes and sex distributions, see Table 1). Mothers were between 22 and 48 years old (*M* = 38.13 years, *SD* = 3.50 years) when they gave birth. All families were white. Of the 244 initially recruited mothers (*n* = 123 male fetuses and *n* = 121 female fetuses), *n* = 231 were used for data analysis, as for *n* = 2 female samples there was no hormonal data as well as no hand scan for any of the measurement time points, and for *n* = 5 male and *n* = 6 female samples there were no corresponding hand scans for any of the measurement time points, leaving *n* = 118 male samples and *n* = 113 female samples. Of the 244 amniocentesis examinations, *n* = 1 male and *n* = 4 female amniotic samples were missing, however, as hand scans were available, these were used for data analysis. Further, the majority of female fetuses T values (97.4%) were below the limit of detection (0.02 ng/ml) or between the limit of detection and the limit of quantification (0.05 ng/ml) and therefore, the female sample was not included for the respective data analysis including hormonal data.

**Table 1:** Number and age (in months) of participants at T1-T5.

Time Point		Boys	Girls	Total
T1: 5 months	*N*	114	111	225
	*M*	5.40	5.45	5.43
	*SD*	0.29	0.31	0.30
T2: 9 months	*N*	101	91	192
	*M*	9.38	9.36	9.37
	*SD*	0.35	0.39	0.37
T3: 20 months	*N*	86	80	166
	*M*	20.54	20.52	20.53
	*SD*	0.34	0.44	0.39
T4: 40 months	*N*	80	78	158
	*M*	40.51	40.56	40.53
	*SD*	0.49	0.65	0.57
T5: 70 months	*N*	73	74	147
	*M*	70.69	70.68	70.69
	*SD*	0.92	1.38	1.17

For recruitment, mothers gave their written informed consent for the use of the data obtained through amniocentesis as well as for re-contacting for the following measurements. At every following measurement (T1-T5) the parents again gave written informed consent for participation. The longitudinal study was approved by the local Ethics Committee of the Science Faculty of the University of Düsseldorf, Germany.

### Materials

#### Prenatal hormone concentration measurements

Total prenatal T and E levels (in ng/ml) were determined in amniotic fluid from amniocentesis samples of mothers recruited from *Praenatal*.*de* (Düsseldorf, Germany). The amniocentesis examinations were carried out between weeks 14 and 18 of gestation (*M* = 14.78, *SD* = 0.84). Amniotic fluid samples were assayed with ultra-performance liquid chromatography and tandem mass spectrometry (described elsewhere [58, 59]). For data analysis, *n* = 117 male samples could be included.

As discussed by Lutchmaya et al. [43], the ratio between amniotic T and E (amniotic T/E) may have a similar impact on early fetal and postnatal development as amniotic T alone. Therefore, we included this ratio in our analysis. Amniotic T/E was calculated as follows:

$$Amniotic\;T/E=\frac{amniotic\;T\;levels\;\left[\frac{ng}{ml}\right]}{amniotic\;E\;levels\;\left[\frac{ng}{ml}\right]}$$

#### 2D:4D

Both hands of the children were scanned using a FIJUTSI fi-60F image scanner at every time point (T1-T5), as hand scans yield both larger sex differences [8] and a higher measurement precision [60, 61] compared to direct measurements. The freeware program *Autometric* [62] was used to measure the ratio between the second and the fourth digit length (2D:4D). Each digit was measured as the length of the midpoint of the ventral proximal crease to the tip in *pixels* using a 100-dpi monitor (100 *pixels* = 2.54 *cm*). Scans in which the fingertips or the ventral creases were not distinct were excluded as well as scans that could not be used for calculation of 2D:4D (e.g. only the length of the second finger was measurable but not of the fourth finger). Two raters, each blind to the sex of the children, measured all hand scans (max. 10 per child). Intraclass correlations indicated high inter-rater reliabilities (all ICCs > .90). The measurements of the two raters were averaged to increase reliability. 2D:4D was calculated as follows:

$$2D:4D=\frac{length\;of\;the\;second\;digit\;\left[pixels\right]}{length\;of\;the\;fourth\;digit\;\left[pixels\right]}$$

D_[r-l]_.

Additionally, the measure D_[r-l]_ defined as the difference between right and left 2D:4D was computed:

$$D_{\left[r-l\right]}=right\;2D:4D-left\;2D:4D$$

### Statistical analyses

A multilevel linear regression model [63, 64] using the R packages lme4 and lmerTest [65–67] with participants as random effects was used to examine the association between amniotic T as well as amniotic T/E and 2D:4D in boys. Age (months since birth, centralized to the participant mean), amniotic T level (logarithmized to achieve normal distribution, centralized to the grand mean) and week of pregnancy the amniocentesis took place (centralized to the grand mean) were entered as predictors of 2D:4D in the first model; age, amniotic T/E level (logarithmized to achieve normal distribution, centralized to the grand mean) and week of pregnancy the amniocentesis took place were entered as predictors in the second model.

The same multilevel linear regression models [63, 64] with participants as random effects were used to examine the association between amniotic T as well as amniotic T/E and D_[r-l]_ in boys. Multilevel linear regression models with participants as random effects were also used to test whether age, sex, and hand were associated with 2D:4D. The advantage of this analysis is that it accounts for participant heterogeneity as well as missing data (distribution of participants for each measurement time point can be seen in Table 1). Age (months since birth, centralized to the participant mean), sex (first coded as boys = 0, girls = 1, then centralized to the grand mean), and hand (first coded as left = 0, right = 1, then centralized to the participant mean) were entered as predictors of 2D:4D. Knickmeyer, Woolson, Hamer, Konneker, and Gilmore [53] also used mixed models in their longitudinal study of 2D:4D. To follow-up on the association between age and 2D:4D, four additional multilevel linear regression models comparing the influence of age between T1 and T2, T2 and T3, T3 and T4, and T4 and T5 were calculated. Lastly, Pearson correlations of 2D:4D between the different time points were conducted and interpreted as follows: small correlation *r* ≥ .10, moderate correlation *r* ≥ .30, and large correlation *r* ≥ .50 [68].

## Results

### 2D:4D and amniotic T and T/E

**Fig 1:**
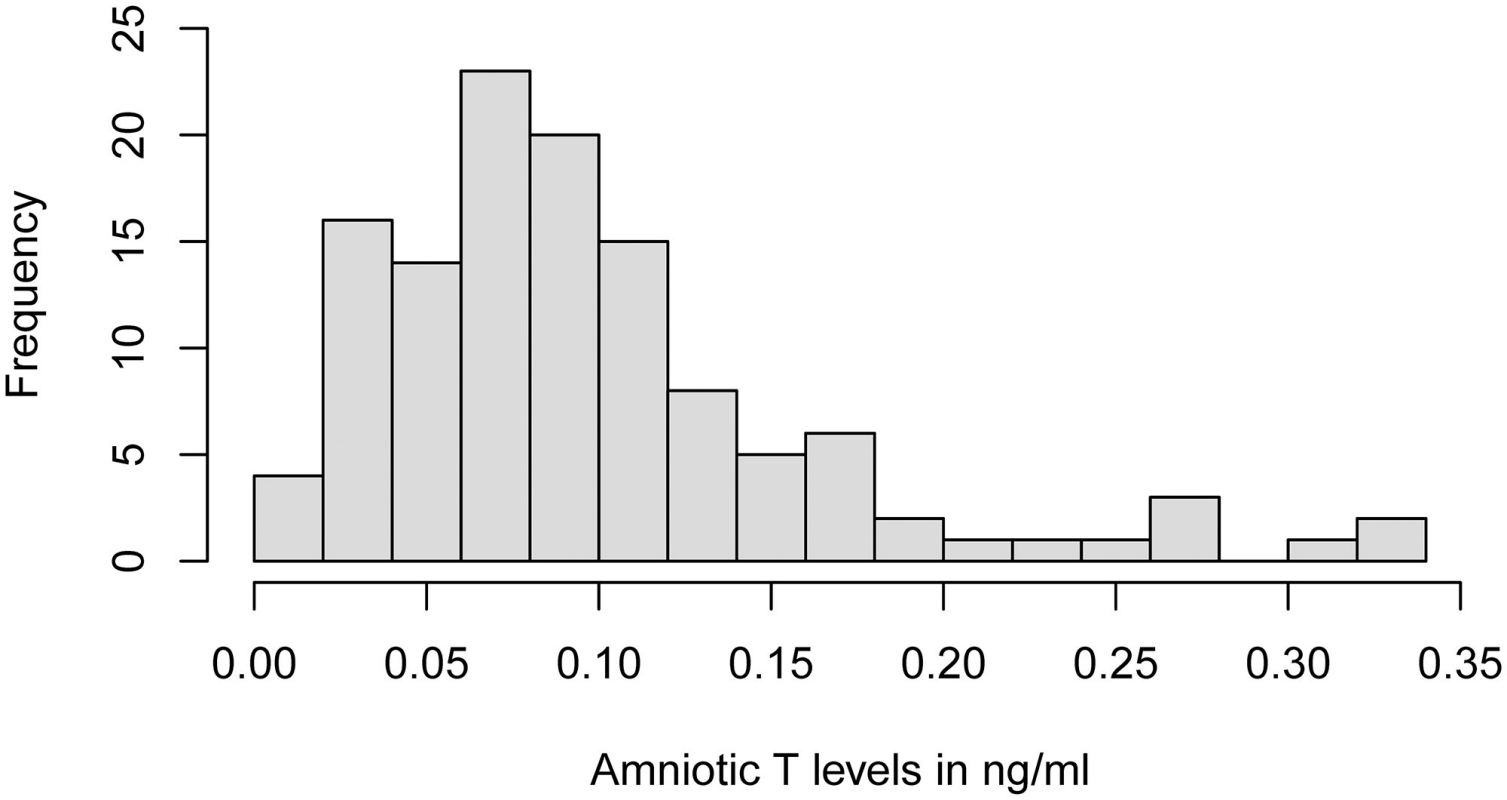
Distribution of male amniotic T-levels (*n* = 117).

Boys had mean T levels of *M* = 0.099 ng/ml (*SD* = 0.065 ng/ml, range: 0.014–0.339 ng/ml, see Fig 1 for distribution) and mean E levels of *M* = 0.146 ng/ml (*SD* = 0.080 ng/ml, range: 0.047–0.496 ng/ml). There were no significant associations between amniotic T or amniotic T/E and 2D:4D, and no interactions with any other factor in boys (controlling for age at measurement of 2D:4D and week of pregnancy of amniocentesis) as indicated by two multilevel linear regression models (see Tables 2 and 3).

**Table 2:** Multilevel linear model with age, amniotic T and week of pregnancy as predictors, and boys’ 2D:4D as outcome.

Effect	Estimate	*SE*	*df*	*t*	*p*
Intercept	0.9329	0.0023	103.3	407.65	< .001
Age***	0.0004	0.0000	500.4	9.34	< .001
Amniotic T	0.0003	0.0036	101.4	0.08	.939
Week of Pregnancy	0.0043	0.0028	103.4	1.54	.127
Age x Amniotic T	0.0001	0.0001	500.4	0.94	.346
Age x Week of Pregnancy	-0.0000	0.0001	500.4	-0.80	.422
Amniotic T x Week of Pregnancy	0.0047	0.0049	129.9	0.96	.339
Age x Amniotic T x Week of Pregnancy	0.0002	0.0001	500.4	1.26	.207

*Note*. Estimates represent unstandardized regression weights. Analyses were performed with the R procedures lme4 and lmerTest with restricted maximum likelihood estimation. Testosterone = logarithmized testosterone level from amniotic fluid; Week of Pregnancy = week of pregnancy of amniocentesis examination; *SE* = Standard error.

***p < .001

**Table 3:** Multilevel linear model with age, amniotic T/E and week of pregnancy as predictors, and boys’ 2D:4D as outcome.

Effect	Estimate	*SE*	*df*	*t*	*p*
Intercept	0.9329	0.0023	103.3	408.41	< .001
Age***	0.0004	0.0000	500.3	9.32	< .001
Amniotic T/E	-0.0014	0.0029	101.7	-0.50	.616
Week of Pregnancy	0.0041	0.0028	104.2	1.45	.149
Age x Amniotic T/E	0.0000	0.0001	500.3	0.56	.573
Age x Week of Pregnancy	-0.0001	0.0001	500.3	-0.94	.349
Amniotic T/E x Week of Pregnancy	0.0034	0.0035	109.7	0.96	.341
Age x Amniotic T/E x Week of Pregnancy	0.0001	0.0001	500.3	1.38	.169

*Note*. Estimates represent unstandardized regression weights. Analyses were performed with the R procedures lme4 and lmerTest with restricted maximum likelihood estimation. *SE* = Standard error.

***p < .001

### D_[r-l]_ and amniotic T and T/E

There were no significant associations between amniotic T or amniotic T/E and D_[r-l]_, and no interactions with any other factor in boys (controlling for age at measurement of D_[r-l]_ and week of pregnancy of amniocentesis) as indicated by two multilevel linear regression models (see Tables 4 and 5).

**Table 4:** Multilevel linear model with age, amniotic T and week of pregnancy as predictors, and boys’ D_[r-l]_ as outcome.

Effect	Estimate	*SE*	*df*	*t*	*p*
Intercept	0.0002	0.0028	102.0	0.06	.956
Age^†^	0.0002	0.0001	157.8	1.95	.053
Amniotic T	0.0038	0.0043	88.9	0.87	.388
Week of Pregnancy	-0.0015	0.0034	104.9	-0.43	.671
Age x Amniotic T	-0.0001	0.0001	157.8	-0.58	.564
Age x Week of Pregnancy	-0.0002	0.0001	157.8	-1.29	.198
Amniotic T x Week of Pregnancy	0.0069	0.0067	101.4	1.03	.305
Age x Amniotic T x Week of Pregnancy	-0.0003	0.0002	157.8	-1.42	.159

*Note*. Estimates represent unstandardized regression weights. Analyses were performed with the R procedures lme4 and lmerTest with restricted maximum likelihood estimation. Testosterone = logarithmized testosterone level from amniotic fluid; Week of Pregnancy = week of pregnancy of amniocentesis examination; *SE* = Standard error.

^†^*p* < .10

**Table 5:** Multilevel linear model with age, amniotic T/E and week of pregnancy as predictors, and boys’ D_[r-l]_ as outcome.

Effect	Estimate	*SE*	*df*	*t*	*p*
Intercept	0.0003	0.0028	100.9	0.10	.923
Age^†^	0.0002	0.0001	156.9	1.94	.054
Amniotic T/E	-0.0036	0.0034	95.5	-1.03	.304
Week of Pregnancy	-0.0011	0.0034	103.0	-0.31	.759
Age x Amniotic T/E	0.0000	0.0001	156.9	0.24	.810
Age x Week of Pregnancy	-0.0001	0.0001	156.9	-1.18	.240
Amniotic T/E x Week of Pregnancy	0.0034	0.0045	107.8	0.75	.453
Age x Amniotic T/E x Week of Pregnancy	-0.0001	0.0002	156.9	-0.74	.463

*Note*. Estimates represent unstandardized regression weights. Analyses were performed with the R procedures lme4 and lmerTest with restricted maximum likelihood estimation. *SE* = Standard error.

^†^*p* < .10

### Temporal stability of 2D:4D

**Fig 2:**
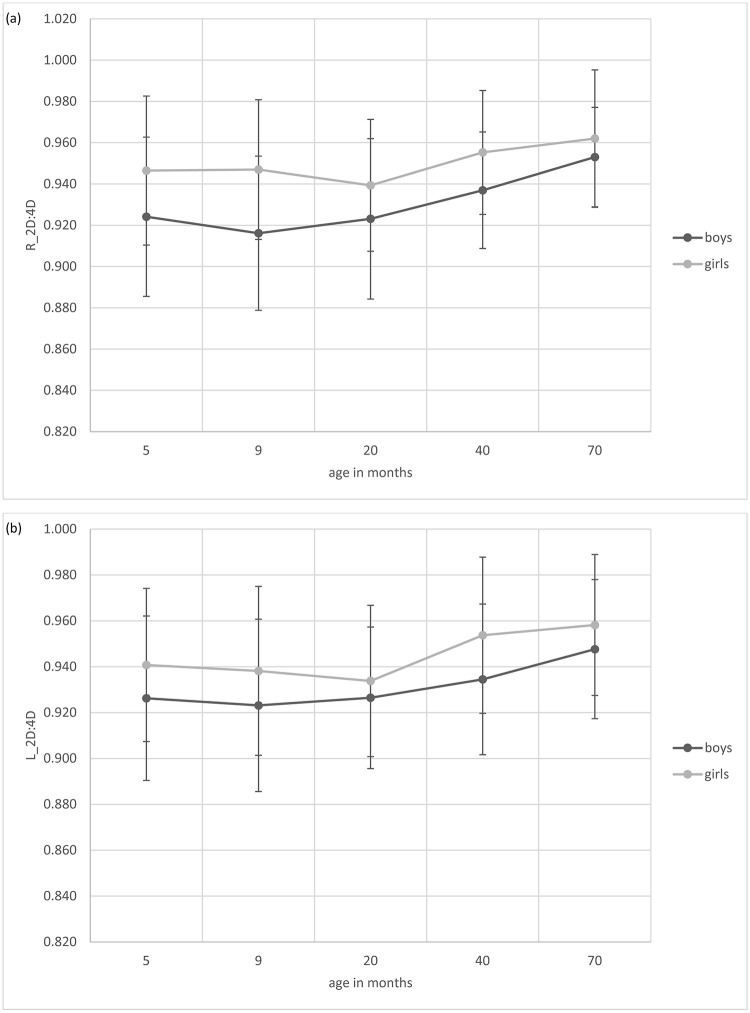
Means and Standard Deviations of (a) Right and (b) Left 2D:4D for every Time Point in Girls and Boys.

Girls had larger 2D:4D compared to boys at every measurement time point. On a descriptive level, boys’ 2D:4D decreased from T1 to T2 and increased from T2 to T3, T3 to T4 and T4 to T5; in girls, right 2D:4D increased whereas left 2D:4D decreased from T1 to T2, 2D:4D of both hands decreased from T2 to T3, and showed an increase from T3 to T4 and T4 to T5 (see Table 6 and Fig 2). The multilevel linear regression model with age, sex, and hand as predictors of 2D:4D (see Table 7) showed a significant main effect of sex. Moreover, there was a significant main effect of age indicating that 2D:4D increased with age. The interaction between sex and age was marginally significant (*p* = .050) and the three-way interaction between sex, age, and hand was significant (*p* = .036). Follow-up analyses on these interactions revealed that a) the effects of age and sex were significant in both hands (all *p* < .001), b) in the right hand, the effect of age was stronger in boys compared to girls (*p* = .006), and c) in the left hand, the effect of age was of equal size for boys and girls (*p* = .761). There was no main effect of hand, no interaction between sex and hand, and no interaction between age and hand. The additional multilevel linear regression models to follow up on the effect of age showed that age was marginally associated with 2D:4D between T1 and T2, but not between T2 and T3. There were significant age effects between T3 and T4, as well as between T4 and T5 (see Table 8). Table 9 indicates that the sex effect remained stable for each measurement time point separately. There was no main effect for hand and no significant interaction between hand and sex for each measurement point separately (see Table 9).

**Table 6:** Sample sizes, means and standard deviations for right and left 2D:4D at T1-T5.

		Right hand			Left hand		
		*N*	*M*	*SD*	*N*	*M*	*SD*
T1	Boys	71	.924	.039	70	.926	.036
	Girls	65	.946	.036	85	.941	.033
	Overall	136	.935	.039	155	.934	.035
T2	Boys	56	.916	.037	68	.923	.038
	Girls	49	.947	.034	60	.938	.037
	Overall	105	.931	.039	128	.930	.038
T3	Boys	51	.923	.039	50	.926	.031
	Girls	51	.939	.032	50	.934	.033
	Overall	102	.931	.036	100	.930	.032
T4	Boys	56	.937	.028	60	.934	.033
	Girls	65	.955	.030	67	.954	.034
	Overall	121	.947	.030	127	.945	.035
T5	Boys	73	.953	.024	72	.948	.030
	Girls	74	.962	.033	74	.958	.031
	Overall	147	.958	.029	146	.953	.031

*Note*. Age at T1: 5 months, at T2: 9 months, at T3: 20 months, at T4: 40 months, and at T5: 70 months.

**Table 7:** Multilevel linear model with sex, age and hand as predictors, and 2D:4D as outcome.

Effect	Estimate	*SE*	*df*	*t*	*p*
Intercept	0.9402	0.0016	212.0	577.57	< .001
Sex***	0.0138	0.0033	212.0	4.24	< .001
Age***	0.0004	0.0000	1025.0	11.72	< .001
Hand	0.0017	0.0015	1025.0	1.08	.280
Sex × Age^†^	-0.0001	0.0001	1025.0	-1.96	.050
Sex × Hand	0.0041	0.0031	1025.0	1.33	.185
Age × Hand	0.0001	0.0001	1039.0	1.01	.311
Sex × Age × Hand*	-0.0003	0.0001	1039.0	-2.10	.036

*Note*. Estimates represent unstandardized regression weights. Analyses were performed with the R procedures lme4 and lmerTest with restricted maximum likelihood estimation. For sex, 0 = boys, 1 = girls. For hand, 0 = left, 1 = right. *SE* = Standard error.

^†^*p* < .10,

*p < .05,

***p < .001

**Table 8:** Multilevel linear model with age as predictor and 2D:4D as outcome.

Measurement		Estimate	*SE*	*df*	*t*	*p*
T1 vs. T2^†^	Intercept	0.9331	0.0021	219.5	447.44	< .001
	Age	-0.0013	0.0008	326.5	-1.71	.089
T2 vs. T3	Intercept	0.9307	0.0021	177.8	445.85	< .001
	Age	0.0002	0.0003	253.5	0.45	.655
T3 vs. T4***	Intercept	0.9392	0.0021	171.7	455.06	< .001
	Age	0.0008	0.0001	279.7	5.36	< .001
T4 vs. T5***	Intercept	0.9504	0.0020	167.6	484.25	< .001
	Age	0.0004	0.0001	373.7	5.35	< .001

*Note*. Estimates represent unstandardized regression weights. Analyses were performed with the R procedures lme4 and lmerTest with restricted maximum likelihood estimation. For sex, 0 = boys, 1 = girls. For hand, 0 = left, 1 = right. T1 = 5 months (4.63–6.87 months), T2 = 9 months (7.59–10.25 months), T3 = 20 months (18.56–21.65 months), T4 = 40 months (38.28–42.64 months), T5 = 70 months (67.16–74.52 months). *SE* = Standard error.

^†^*p* < .10,

***p < .001

**Table 9:** Multilevel linear model with hand and sex as predictors and 2D:4D as outcome for each measurement time point.

Measurement		Estimate	*SE*	*df*	*t*	*p*
T1	Intercept	0.9348	0.0024	181.9	388.51	< .001
	Hand	0.0052	0.0035	120.2	1.50	.135
	Sex***	0.0188	0.0048	182.0	3.90	< .001
	Hand x Sex	0.0024	0.0069	120.2	0.35	.731
T2	Intercept	0.9303	0.0027	140.4	346.95	< .001
	Hand	-0.0019	0.0045	84.6	-0.43	.671
	Sex***	0.0213	0.0054	140.4	3.96	< .001
	Hand x Sex	0.0148	0.0089	84.6	1.65	.102
T3	Intercept	0.9307	0.0025	119.2	379.46	< .001
	Hand	-0.0013	0.0053	81.6	-0.24	.809
	Sex*	0.0115	0.0049	119.2	2.35	.021
	Hand x Sex	0.0170	0.0106	81.6	1.60	.113
T4	Intercept	0.9456	0.0023	139.1	408.97	< .001
	Hand	0.0025	0.0033	108.4	0.76	.451
	Sex***	0.0173	0.0046	139.8	3.74	< .001
	Hand x Sex	-0.0044	0.0066	108.4	-0.67	.507
T5	Intercept	0.9554	0.0021	144.1	462.26	< .001
	Hand	0.0042	0.0027	143.3	1.57	.119
	Sex*	0.0096	0.0041	144.1	2.32	.022
	Hand x Sex	-0.0009	0.0054	143.3	-0.17	.864

*Note*. Estimates represent unstandardized regression weights. Analyses were performed with the R procedures lme4 and lmerTest with restricted maximum likelihood estimation. For sex, 0 = boys, 1 = girls. For hand, 0 = left, 1 = right. T1 = 5 months (4.63–5.43 months), T2 = 9 months (7.59–10.25 months), T3 = 20 months (18.56–21.65 months), T4 = 40 months (38.28–42.64 months), T5 = 70 months (67.16–74.52 months). *SE* = Standard error.

*p < .05,

***p < .001

Regarding the reliability of 2D:4D between measurement time points, Pearson correlations revealed significant positive correlations for the right hand between T1 and T2, *r* = .42, *p* = .001, between T2 and T3, *r* = .29, *p* = .037, between T3 and T4, *r* = .50, *p* < .001, and between T4 and T5, *r* = .66, *p* < .001. For the left hand, significant positive correlations could be found between T1 and T2, *r* = .47, *p* < .001, between T2 and T3, *r* = .30, *p* = .023, between T3 and T4, *r* = .39, *p* = .001, and between T4 and T5, *r* = .61, *p* < .001.

## Discussion

The current study used a longitudinal design and had two main aims: (1) to determine whether 2D:4D is associated with prenatal androgen (T and T/E ratio) concentrations measured from amniotic fluid obtained between 14 and 18 weeks of gestation, and (2) to examine the stability of sex differences in 2D:4D at 5, 9, 20, 40, and 70 months of age. There was no significant correlation between 2D:4D respectively D_[r-l]_ and either amniotic T or the T/E ratio in boys for any time point. For girls, amniotic T and T/E ratio could not be determined and therefore no analysis was performed. At each time point, a consistent sex effect was revealed with girls showing larger 2D:4D ratios than boys. Age was associated with 2D:4D, with post-hoc-analyses revealing a marginally significant decrease in 2D:4D from 5 to 9 months of age and statistically significant increases in 2D:4D from 20 to 40 and from 40 to 70 months of age. Significant positive correlations for 2D:4D between different time points suggest a moderate level of stability during infancy and childhood. However, when taken together, the results of this study indicate that 2D:4D exhibits considerable lability during infancy and also question the validity of 2D:4D as a marker of prenatal androgen exposure.

The current state of research does not allow precise conclusions about the hypothesis that 2D:4D is a marker of prenatal androgen action, with heterogeneity in study designs and differing results providing an inconsistent picture. In our study, we did not find any associations between 2D:4D respectively D_[r-l]_ and either amniotic T or T/E in boys for any of the postnatal time points. However, the absence of female amniocentesis data is especially unfavorable. This is because it precluded the opportunity to try to replicate the finding of Ventura et al. [48] of a significant negative association between T levels and left 2D:4D in girls (but not boys). Ventura et al. [48] hypothesized that because boys are already exposed to higher prenatal T levels than girls, increased T above the average might not have a similar impact on male compared to female 2D:4D, and so may potentially account for the non-significant correlation in boys. On the other hand, Lutchmaya et al. [43] found a statistically significant negative association between the T/E ratio and 2D:4D that remained even when controlling for sex. The attempted replication of Lutchmaya et al. [43] by Richards et al. [44] did not find any of the hypothesized correlations for either sex. This was despite the higher statistical power associated with there being more than twice as many mother-child-dyads as in the original study. These differences in findings may stem from a bias concerning the reliability of data obtained through amniocentesis. Notably, amniocentesis samples reflect only a small time window during prenatal development. Lutchmaya et al. [43] reported that the examinations took place during the second trimester of pregnancy without specifying the mean and range of gestational weeks. Other studies in this area examined amniocentesis samples collected between 15 and 22 weeks [44], 16 and 24 weeks (*M* = 17.2 [48]), and 14 and 18 weeks (*M* = 14.78; current study). This may account for the differing results, as literature suggests considerable fluctuations of T during the second trimester as well as a peak around week 17 [35, 36]. A more recent study of Kuijper, Ket, Caanen, and Lambalk [69] reports that T levels seem to increase between weeks 14 and 22, with no clear peak for boys and a peak around week 18 for girls. Due to risks and ethical considerations, repeated sampling of amniotic fluid in humans is not possible. Nevertheless, amniocentesis data may allow the most precise information available to assess the hormonal intrauterine environment of a developing human fetus [33]. As Hollier, Keelan, Hickey, Maybery, and Whitehouse [70] discussed, to date there is no “gold standard” to examine hormone levels to which the fetus is exposed during pregnancy. Other studies have revealed inconsistent and mostly non-significant associations between children’s 2D:4D and alternative measures like maternal plasma or cord blood samples to assess T levels in pregnant women [71–74].

Another point to consider is that prenatal androgens are unlikely to be the only determining factor for 2D:4D as the sex difference in prenatal androgen levels is much larger than the sex difference observed for 2D:4D [11, 75]. Furthermore, studies of individuals with CAIS show differences in 2D:4D despite T having no physiologic effect [11, 12]. However, Zheng and Cohn [76] showed that active signaling and density of androgen and estrogen receptors differ in the second and fourth digit, which may lead to the sexually differentiated pattern of 2D:4D (female > male) in a mouse model. The sex difference in 2D:4D in mice was present as early as the 17th day of embryonic life [76]. A direct manipulation of the intrauterine environment of mice showed that an activation or blocking of the androgen receptor resulted in changes of the total paw and digit length as well as 2D:4D [77]. That is, an activation of the androgen receptor led to an increase of 2D:4D in males, whereas a blocking of the androgen receptor led to a decrease of 2D:4D in females [77]. This finding is also in line with Swift-Gallant, Di Rita, Coome, and Monks [78] who found an increase in 2D:4D in mice with global androgen receptor overexpression compared to wildtype mice and mice with neural-specific androgen receptor overexpression regardless of sex. Other studies administered T directly as an intramuscular injection into the dam and examined 2D:4D in the offspring. Talarovičová, Kršková, and Blažeková [79] found a significant effect of T administration on the length of the second and fourth digit as well as on 2D:4D. Administration of T, compared to sesame oil as a control, led to a lengthening of the fourth digit, a shortening of the second digit, and a smaller 2D:4D. Contrary, another study found shortened second and fourth digits in the offspring after T (respectively T combined with flutamide) administration compared to a control group that was supplemented with olive oil only, however, no effect of T administration on 2D:4D [80]. These findings indeed suggest an influence of androgens on digit lengths and 2D:4D but also reveal the complex interplay of hormones, chromosomes, and gonads to determine the sex and sex-specific characteristics of organisms and the influence on digit growth.

Despite the current study revealing stable sex differences with girls exhibiting larger 2D:4D compared to boys, 2D:4D appears unable to reliably discriminate between the sexes as the magnitude of 2D:4D in girls and boys reveals a large overlap. This could be shown in various other studies reporting the expected pattern of 2D:4D, however, without significant sex differences and/or only small effect sizes [50–53, 81]. Furthermore, girls show considerable variability in 2D:4D yet relative homogeneity in amniotic T levels [36, 44]; this was also true for the current study, as 97.4% of girls’ T levels were under the limit of detection or quantification. Another observation that questions the validity of 2D:4D is its association with age in young cohorts, an effect reported not only in the current study but also in various others within the literature [51–55, 81, 82]. The significant effect of age indicates that 2D:4D continues to develop during childhood. Importantly, the reliability of 2D:4D at this time is also questionable, as the current study observed only moderately sized correlations between time points. Likewise, the association between age and 2D:4D makes the results of Lutchmaya et al. [43] and Richards et al. [44] difficult to compare, as 2D:4D was measured at 2 and 4.5 years of age respectively.

Notably, research does not support reliable sex differences of 2D:4D in younger cohorts, as noted in another study of our group [49], and it appears that sex differences become larger and more stable during adolescence and adulthood [8, 20, 50–52]. Prepubertal girls and boys show nearly the same concentrations of fluctuating sex hormones [83]. There is only a short period of time during the first 6 months after birth in which specifically T levels increase in boys, whereas the levels do not differ to a greater extent between girls and boys until the onset of puberty [84, 85]. A comparison of characteristics that can be associated with the influence of prenatal sex hormones should be highly reliable between these two time points—birth and puberty. However, studies failed to replicate a stable sex difference in young cohorts [56, 86], questioning the assumption that prenatal amniotic T levels explain a significant proportion of variance in 2D:4D. The measurement of digit lengths in young cohorts compared to adult cohorts may face other methodological issues that challenge the reliability and validity of the measurement itself. Young infants show a much higher proportion of body fat tissue compared to adults [87], which may specifically impair indirect measurement techniques like hand scans, as hand creases tend to shift more in between different measurements when pressed onto the scanner glass. Furthermore, very young children and infants may be less likely to cooperate in the measurement procedure like adults normally do. To control for this, another more direct measurement technique may be a better approach to examine 2D:4D in young cohorts and, compared to indirect measurement techniques, would also yield greater effect sizes [88]. In fact, McIntyre, Ellison, Lieberman, Demerath, and Towne [51] and McIntyre, Cohn, and Ellison [52] used radiographs in their cohorts aged 1 month up to 18 years. However, they found only low to moderate differences in 2D:4D between males and females with more stable differences emerging with age. The lack of comparability between different studies in terms of measurement time point and measurement technique impairs the interpretation of different results specifically in prepubertal cohorts.

Lastly, there are some limitation of the current study that should be addressed. It has already been discussed that the timing of the amniocentesis examination may account for different results between studies. Further, there is a relative large interval between human limb development and amniocentesis examinations as the human limb development starts much earlier, approximately during week 4 of gestation and by the end of week 8 of gestation the upper and lower limbs are already mostly developed [89, 90], compared to amniocenteses which are usually performed around week 15^+0^ of gestation [91]. Therefore, prenatal hormone levels obtained through amniotic sampling may not fully reflect the intrauterine environment during limb development. Additionally, amniotic fluid only reflects the hormonal environment the fetus is subjected to, not the actual hormonal concentrations of the fetus [69]. Nevertheless, amniotic fluid sampling may be the best method available to obtain information about fetal hormonal environment, due to obvious ethical considerations. Also the 2D:4D digit ratio is facing important influencing factors like the number and order of siblings and their sex (however, with contradictory results, see Králík, Hupková, Zeman, Hložek, Hlaváček, Slováčková, et al. [92] and Saino, Leoni, and Romano [93]). Further, as it is hypothesized that 2D:4D differs between different ethnic groups [94, 95], the current results are not generalizable as they only refer to the German and solely white population.

The motivation to obtain reliable and valid information about the intrauterine hormonal environment by simply measuring digit lengths is understandable considering the difficulties associated with obtaining more direct measurements. The literature does provide some evidence for 2D:4D as it exhibits a moderate sized sex difference as well as associations with other sexually differentiated characteristics. However, the evidence that 2D:4D reflects prenatal androgen action is more limited and a comparison of available studies is difficult. Therefore, 2D:4D does not seem to be a feasible proxy for intrauterine androgen exposure. Although the sex difference in 2D:4D shows a good replicability, especially in adult samples, the hypothetical fundament of its origins is not sufficiently examined. If 2D:4D shall be used as a marker for prenatal androgen action it is necessary to examine the developmental basis. Further, instead of conducting novel research on associations between 2D:4D and various other characteristics, research should focus on replications with highly comparable study designs and on testing the underlying hypothesis that 2D:4D is a valid marker of intrauterine androgen action.

## Supporting information

S1 Fig Data inclusion and exclusion chart. (TIF)
