# Supplementary figures and images for: The association of prenatal amniotic sex hormones and digit ratio (2D:4D) in children aged 5 to 70 months: A longitudinal study
Source: PLoS One. 2023 Mar 23;18(3):e0282253. doi: 10.1371/journal.pone.0282253 (PMC10035896; doi:10.1371/journal.pone.0282253)

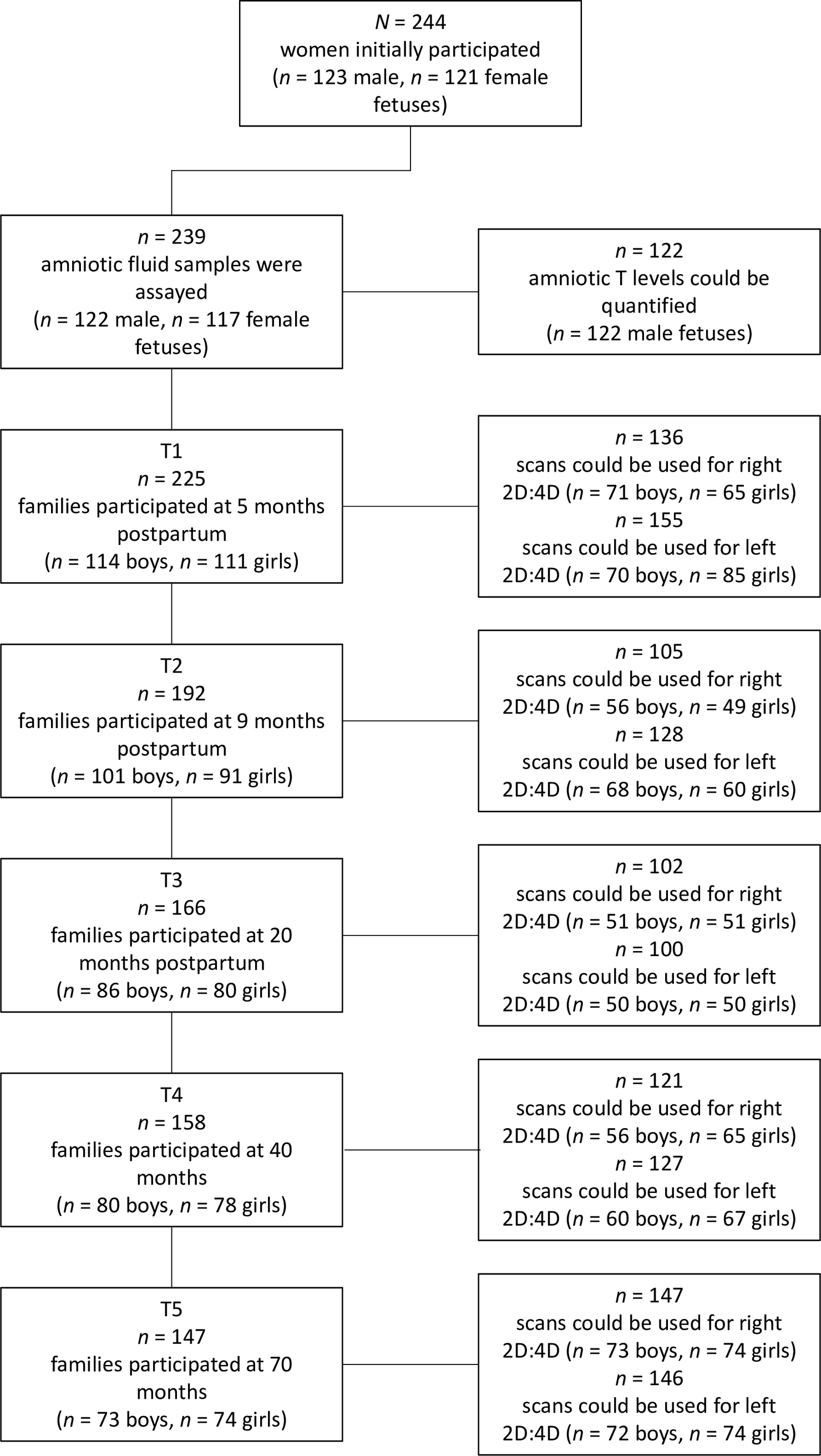

Supplement: S1 Fig — (TIF) [file pone.0282253.s001.tif]
